# Suicide behaviour and arsenic levels in drinking water: a possible association? A review of the literature about the effects of arsenic contamination in drinking water on suicides

**DOI:** 10.1186/s41935-017-0005-y

**Published:** 2017-07-18

**Authors:** Gianmarco Troiano, Isabella Mercurio, Paola Melai, Nicola Nante, Massimo Lancia, Mauro Bacci

**Affiliations:** 1 Public Health University of Siena, Siena, Italy; 2 Forensic Medicine, Department of Medical Sciences, Surgical and Neurosciences, Hospital Santa Maria Alle Scotte, Siena, Italy; 3 Hospital of Perugia, Perugia, Italy; 4 Section of Legal Medicine, Forensic Science and Sports Medicine, University of Perugia, Perugia, Italy; 5 Department of Molecular and Developmental Medicine, Area of Public Health, University of Siena, 2 Via A. Moro, Siena, 53100 Italy

**Keywords:** Arsenic, Suicides, Drinking water, Intoxication, Review, Environmental health, Mental health

## Abstract

**Background:**

A considerable part of the global population is exposed to arsenic-contaminated drinking water which is the main source of inorganic arsenic(As) exposure in humans. Arsenic exposure interferes with the action of enzymes, essential cations, and transcriptional events in cells throughout the body, and a multitude of multisystemic non-cancer effects might ensue. The aim of our review was to evaluate the effects of arsenic contamination in drinking water on suicides rates.

**Methods:**

A systematic literature search (English written literature) was conducted in electronic databases MEDLINE, SCOPUS. Evidences dating from 1999 till 2016 have been collected. A manual search of reference lists of included studies and review articles was successively performed. All references of the retrieved studies were also reviewed to avoid missing relevant publications.

The key search terms included: “arsenic AND water AND suicide”.

**Results:**

The literature search yielded 13 publications, but we identified 2 manuscripts available for this systematic review. The 2 studies included in the review, were published in 2015 and in 2017 and settled in Italy and Hungary. The levels of arsenic in the waters ranged from 0.016 μg/l to >50 μg/l. The findings of the two studies are conflicting, in fact Pompili et al. reported an apparently beneficial effect of arsenic on suicides rates, with an inverse correlation of arsenic concentration and local suicide rates, in contrast to a positive correlation with natural-cause mortality rates.

**Conclusions:**

Our review led to conflicting results, so the diatribe about the real effects of arsenic intake of suicidal behaviors is still open. Therefore, we encourage other colleagues to conduct further studies in other locations in order to have more reliable results.

## Background

Suicide is a complex phenomenon and represents a major worldwide health problem that encourages searches for factors that may contribute to or limit its risk. Worldwide suicide rates average 11.4/100,000/year; in Europe, the rates range from 4.9 in Greece to 25.4 in Hungary, while in Italy it is almost 7.5/100,000 (WHO 2016; Isabella Mercurio et al. 2016).

A considerable part of the global population (e.g., residents of some parts of Argentina, Bangladesh, Chile, China, Hungary, India, Mexico, Taiwan and the USA) is exposed to arsenic-contaminated drinking water which is the main source of inorganic arsenic(As) exposure in humans (Rihmer et al. 2015; Stefania Milione et al. 2016).

Arsenic is found widely in the environment, notably in pesticides and wood-preservatives, it could be found at high concentrations in some foodstuffs, and is highly toxic in a concentration- or dose-dependent manner (De Vivo et al. 2009; Eleonora Ricchi et al. 2016; Mercurio et al. 2016).

The carcinogenic role of arsenic compounds was first noted over 100 years ago in the Hutchinson (1887) observation that an unusual number of skin tumors developed in patients treated with arsenicals. In a 1980 review of arsenic, the International Agency for Research on Cancer (IARC 1980) determined that inorganic arsenic compounds are skin and lung (via inhalation) carcinogens in humans. Since 1980, several additional studies of cancer and exposure to arsenic in drinking water have been completed (National Research Council (US) Subcommittee on Arsenic in Drinking Water 1999).

Arsenic exposure interferes with the action of enzymes, essential cations, and transcriptional events in cells throughout the body, and a multitude of multisystemic non-cancer effects might ensue. These selected non-cancer effects derived from chronic ingestion of arsenic in drinking water and are the most relevant for the effects on health (National Research Council (US) Subcommittee on Arsenic in Drinking Water 1999).

Studies in Bangladesh and in the United States reported that people with higher arsenic contamination suffered more from depression (Zierold et al. 2004). Furthermore, a cross-sectional study in two villages in Inner Mongolia, China, found that the mental health of the subjects in the arsenic-affected village was worse than in those in the arsenic-free village (OR = 2.5, 95% CI = 1.1.–6.0) (Fujino et al. 2004; Nante et al. 2016).

Other studies have shown that mental health problems (e.g. depression) are more common among the people affected by arsenic contamination (Brinkel et al. 2009).

The aim of our study, was therefore, to analyze all the available literature about this topic through a systematic search in order to understand the possible effects that arsenic contamination of drinking water could have on suicides rates.

## Methods

A systematic literature search (English written literature) was conducted in electronic databases MEDLINE, SCOPUS. Evidences dating from 1999 till 2016 have been collected. A manual search of reference lists of included studies and review articles was successively performed. All references of the retrieved studies were also reviewed to avoid missing relevant publications.

The key search terms included: “arsenic AND water AND suicide”.

After full screening of titles, abstracts and full texts, the selection of included studies was based on the availability of information about the association of arsenic levels in drinking water and suicides.

Studies were selected in a 2-stage process. Titles and abstracts from electronic searches were scrutinized by 2 reviewers independently (I.M. and G.T.) and full manuscripts and their citations list were analyzed by a third reviewer (P.M.) to retrieve missing articles and to select the eligible manuscript according to the inclusion and the exclusion criteria. The level of agreement between the reviewers was high.

## Results

**Fig. 1:**
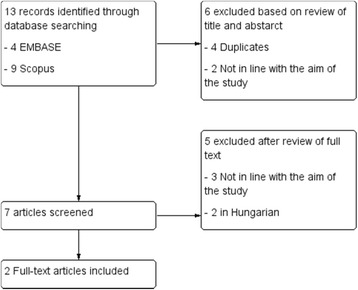
Flow diagram for identifying studies included in the review

The literature search yielded 13 publications. The titles and abstracts of these manuscripts were screened, resulting in 7 studies considered potentially eligible to be included in the review. Of the total of 7 identified relevant manuscripts, 3 studies were excluded after the examination of the full text; 2 were excluded because they were written in Hungarian. Finally, we identified 2 manuscripts available for this systematic review (Rihmer et al. 2015; Pompili et al. 2017) (Fig. 1).

The 2 studies included in the review (Table 1), were published in 2015 and in 2017 and settled in Italy and Hungary. The levels of arsenic in the waters ranged from 0.016 μg/l (Pompili et al.) to >50 μg/l (Rihmer et al.). The findings of the two studies are conflicting, in fact Pompili et al. reported an apparently beneficial effect of arsenic on suicides rates, with an inverse correlation of arsenic concentration and local suicide rates, in contrast to a positive correlation with natural-cause mortality rates.

**Table 1:** Main characteristics of the studies included in the review

Author, year	Setting	Study period	Study population	Arsenic levels	Suicide rates (N/100,000 deaths)
Rihmer, 2015	Hungary	2005-2011	1639 settlements divided in: (I) 5871^a^ (II)5017^a^ (III)3066^a^ (IV)4373^a^	(I) Low 10 μg/l (II) Intermediate 11–30 μg/l (III) High 31–50 μg/l (IV) Very high >50 μg/l	(I) – 23.6 (18.4) (II) – 30.6 (17.2) (III) – 32 (17.7) (IV) – 33.8 (21.4) *P* < 0.001
Pompili, 2017	Italy	−1980–2011 (analysis of suicides rates) −2009–2010 (collection of water samples)	145 sites 157 water samples 104 provinces	0.969 μg/l CI 0.543–1.396 μg/l	7.53^b^

^a^average population of settlements in the group; division based on arsenic levels; ^b^Negatively associated with suicide rates, calculated through univariate regression modeling

## Discussion

Data derived from population-based studies, clinical-case series, and case reports relating to the ingestion of inorganic arsenic in drinking water, medications, or contaminated food or beverages showed the capacity of arsenate and arsenite to adversely affect multiple-organ systems. The clinical appearance of the non-cancer manifestations of arsenic intoxication in humans is dependent on the magnitude of the dose and the time course of exposure. Although the toxicokinetic and toxicodynamic interaction between those two measures has not been well characterized, several general findings emerge from the available data (National Research Council (US) Subcommittee on Arsenic in Drinking Water 1999).

The Agency for Toxic Substances and Disease Registry stated that acute toxic exposures to inorganic arsenic have been shown to lead to emotional lability and memory loss. A mechanism of action has not been identified, but perhaps long-term exposure to arsenic may interfere with the neurotransmitters associated with depression. Mechanistic research into effects on the brain and mental development is needed to understand the role that arsenic may play in the development of neurological disease ((ATSDR) AfTSaDR 2000).

The findings of the two studies included in our review are conflicting: in fact Pompili et al., contrariwise to the results of Rihmer et al., reported an apparently beneficial effect of arsenic on suicides rates, with an inverse correlation of arsenic concentration and local suicide rates, in contrast to a positive correlation with natural-cause mortality rates.

The studies included in our review have some limitations: the preliminary study conducted by Rihmer used aggregated (i.e., non-individual) data so the results and conclusions are not inevitably true on the level of individuals; they didn’t adjust the model for important medical and socio-demographic determinants of suicidal behavior and they had no data on differences in bottled water consumption between settlements. Since this was the first preliminary report on the possible association between arsenic intake from drinking water and suicidal behavior the results need further confirmations by studies preferably with prospective design and based on individual-level arsenic data. Such studies would be also able to decide whether there is a causal link behind the association found by the authors (Rihmer et al. 2015).

The study conducted by Pompili et al., instead, is an ecological study and it is subject to the limitations inherent ecological designs, thus cannot directly link individual exposures and effects. The exploratory nature of analyses, and the unexpected outcomes observed, though consistent between the sexes and over times sampled, do not support certain conclusions or generalizations. The authors were unable to control possible confounding factors, such as natural processes, effects of mining or industrial wastes, use of agricultural chemicals, potential contributions of arsenic in foodstuffs, or regional differences in use of bottled water. These findings, however, suggested that exposure to low concentrations of arsenic in drinking water might exert an unexpected, apparent “protective effect” against suicide (Pompili et al. 2017).

## Conclusions

From the systematic research and analysis of all the available literature it was not possible to understand, in a reliable way, the real effect that arsenic contamination of drinking water could have on suicides rates. So the diatribe on this topic is still open: because of the conflicting results reported by the above mentioned studies, we encourage other colleagues to conduct further studies in order to have further, and more reliable, results.
